# Post-pandemic seasonal dynamics of hospitalised COPD exacerbations and aetiologies in the COPD population

**DOI:** 10.1183/23120541.00258-2024

**Published:** 2024-09-30

**Authors:** Hnin Aung, Aye Aung, Hamish J.C. McAuley, Omer Elneima, Cara Flynn, Tracy Thornton, Helen Evans, Christopher E. Brightling, Adam Wright, Neil J. Greening

**Affiliations:** 1 Department of Respiratory Sciences, University of Leicester, Leicester, UK; 2 Institute for Lung Health, NIHR Respiratory Biomedical Research Centre, University Hospitals of Leicester, Leicester, UK

## Abstract

The impact of seasonality on the morbidity due to acute exacerbations of COPD (AECOPD) is well recognised, with winter months typically associated with increased respiratory infections and healthcare utilisation among the COPD population [1]. The first wave of the coronavirus disease 2019 (COVID-19) pandemic saw at least 50% decline in AECOPD admissions associated with fewer viral triggers [2]. However, after relaxation of lockdown measures, it is unknown if there has been a longer-term shift in the seasonal pattern of AECOPD hospitalisations and their specific aetiologies, considering the heterogeneity behind exacerbations. This information may help healthcare providers prepare targeted preventative measures and resource allocation.

## Abstract 2

**This study uncovered a reversal of seasonal variation in hospitalised COPD exacerbation events after the COVID-19 pandemic. Investigation of updated seasonal exacerbation patterns and triggers can help initiate preventive strategies effectively.** https://bit.ly/3wN71h0

*To the Editor:*

The impact of seasonality on the morbidity due to acute exacerbations of COPD (AECOPD) is well recognised, with winter months typically associated with increased respiratory infections and healthcare utilisation among the COPD population [1]. The first wave of the coronavirus disease 2019 (COVID-19) pandemic saw at least 50% decline in AECOPD admissions associated with fewer viral triggers [2]. However, after relaxation of lockdown measures, it is unknown if there has been a longer-term shift in the seasonal pattern of AECOPD hospitalisations and their specific aetiologies, considering the heterogeneity behind exacerbations. This information may help healthcare providers prepare targeted preventative measures and resource allocation.

We conducted an observational study targeting adult COPD patients who were on maintenance inhaled therapy and admitted to Glenfield Hospital, Leicester, UK, a secondary care cardiorespiratory hospital that admits 95% of all acute respiratory admissions due to AECOPD in the region. The objective was to compare the frequency of AECOPD admissions across all seasons during the post-pandemic year from 1 December 2021 to 30 November 2022, and characterise the triggers of each exacerbation at the time of admission. Study approval was granted from the East Midlands Research Ethics Committee (21/EM/0184). At recruitment, exacerbation triggers were categorised as viral, bacterial, eosinophilic and other, as guided by established clusters previously proposed [3]. Events due to viral triggers were detected by positive PCR assay and symptoms suggestive of viral infection (*e.g.* fever or influenza-like symptoms), as well as reported contact with anyone having a viral infection. Bacterial triggers were defined as acquisition of a new strain in sputum culture or at least two of the established criteria (neutrophil to lymphocyte ratio ≥6, C-reactive protein >50 mg·L^−1^ and infiltration on chest radiograph [4, 5]) with negative viral PCR assay. Eosinophilic exacerbations were selected by blood eosinophil count ≥0.3×10^9^ cells·L^−1^ on admission with no signs of acute infection, and other triggers included AECOPD events of unknown aetiologies or mimicked by comorbidities such as pulmonary embolism and heart failure. Data were presented across four seasons. Differences across seasons were compared using Kruskal–Wallis with individual seasons compared *post hoc*. Length of hospital stay (LOHS) between bacterial and eosinophilic admissions were compared using the Mann–Whitney test. Analysis was performed using Stata 18.0 (StataCorp LLC, College Station, TX, USA).

Between 1 December 2021 and 30 November 2022, 196 COPD patients (mean±sd age 68±10 years, 110 (56%) females, mean±sd forced expiratory volume in 1 s of 34±15% predicted, 122 (61%) with cardiovascular comorbidities) who required hospitalisation due to AECOPD were recruited. 109 (56%) received oxygen or noninvasive ventilatory support. Admissions were more frequent in spring and summer (n=68 (34.7%) and n=58 (29.6%), respectively) than in autumn and winter (n=39 (19.9%) and n=31 (15.8%), respectively; spring *versus* winter p<0.0005) (figure 1a). The occurrence of hospitalised AECOPD events varied significantly by season (p=0.004). A significantly higher proportion of viral triggers was observed in summer than in winter (n=18 (37.5%) *versus* n=4 (8.3%), respectively, out of 48 with viral triggers; p=0.0005).

**FIGURE 1:**
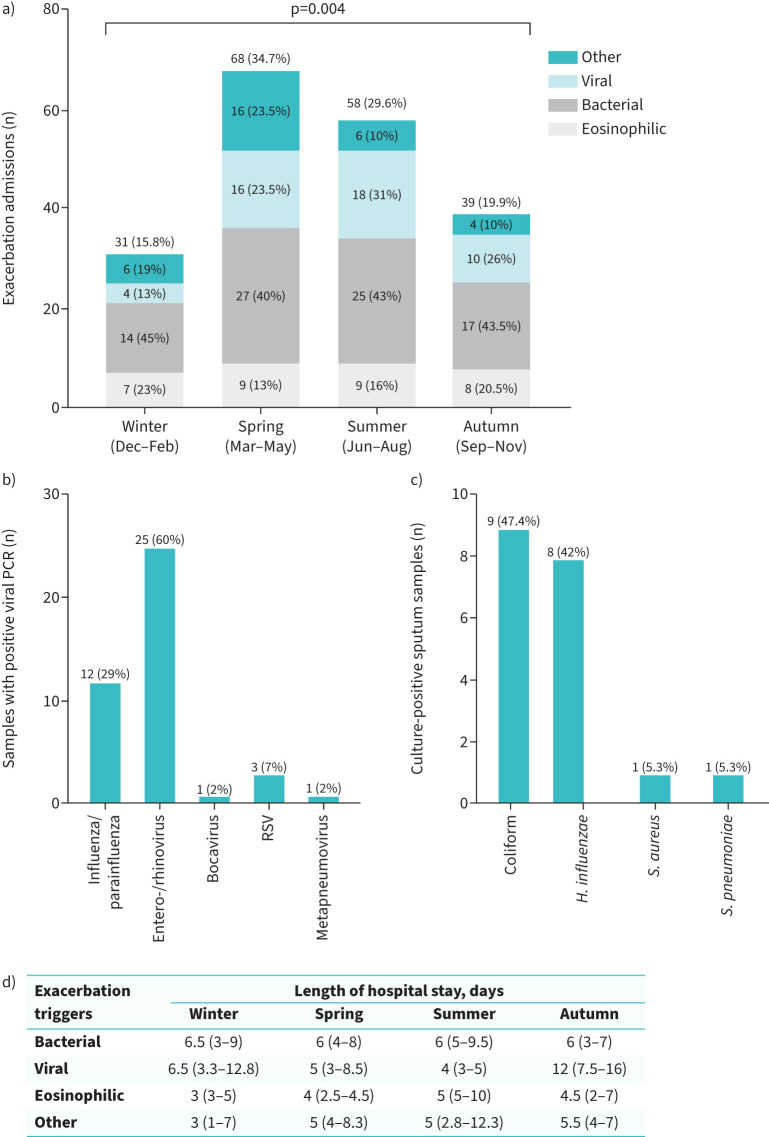
a) Rate of newly hospitalised exacerbations between 1 December 2021 and 30 November 2022, according to seasons (spring, summer, autumn and winter) and triggers (bacterial infection, viral infection, eosinophil-predominant exacerbations and other triggers). b) Frequency of causal viruses among PCR-confirmed viral exacerbation events (n=42). RSV: respiratory syncytial virus. c) Proportions of culture-positive sputum bacterial samples (n=19). *H. influenzae*: *Haemophilus influenzae*; *S. aureus*: *Staphylococcus aureus*; *S. pneumoniae*: *Streptococcus pneumoniae*. d) Subgroup analysis of the length of hospital stay for each type of trigger and each season. Data are presented as median (interquartile range).

Respiratory infections (n=83 (42.4%) bacterial and n=48 (24.5%) viral aetiologies) were the predominant admission triggers, followed by eosinophilic exacerbations (n=33 (16.8%)). Other triggers identified as contributing factors to admissions (n=32 (16.3%)) included heart failure (n=6) and pulmonary embolism (n=1).

170 (87%) participants had nasal swabs taken for extensive viral PCR (79%) or rapid Cepheid Xpert assay (21%) (Cepheid, Sunnyvale, CA, USA). Rhinovirus (n=25 (60%)) was the most common cause among the 42 PCR-confirmed viral exacerbations, followed by influenza/parainfluenza strains (n=12 (29%)), respiratory syncytial virus (n=3 (7%)), and human metapneumovirus and bocavirus (n=1 (2%) for each) (figure 1b). For the 60 (31% of 196) participants tested for sputum bacterial culture, the 19 with isolated species had coliform (n=9 (47.4%)), *Haemophilus influenzae* (n=8 (42%)), *Staphylococcus aureus* (n=1 (5.3%)) and *Streptococcus pneumoniae* (n=1 (5.3%)) (figure 1c).

The median (interquartile range) LOHS for all participants was 5 (3–8) days, with 6 (4–8) days for bacterial triggers, 5 (3–9) days for viral triggers, 5 (3–7) days for other triggers and 4 (3–6) days for eosinophilic exacerbations. Bacterial triggers posed a longer LOHS than eosinophilic exacerbations (p=0.006), but cautious interpretation is warranted due to small sample size and potential confounding factors such as age or comorbidities. The LOHS in different seasons is shown in figure 1d.

This study demonstrated that the seasonal variation of AECOPD admissions following the COVID-19 pandemic had not returned to the expected pattern [1]. The highest admission numbers were in spring and summer, with double the prevalence of viral admissions compared to winter. Our findings echo other recent evidence from the northern hemisphere that indicated a reverse AECOPD seasonality pattern during the pandemic, with 1.5 times higher numbers of severe episodes in summer than winter [6]. Importantly, our data suggest that the trend persists far beyond the initial pandemic impact.

Infection triggers caused 67% of AECOPD hospitalisations, resulting in the longest LOHS of all causes, aligning with existing evidence [7]. Conversely, admissions with blood eosinophilia, typically 10–40% of COPD admissions [8], caused the shortest LOHS, highlighting a negative association of LOHS and blood eosinophil count ≥0.3×10^9^ cells·L^−1^ at admission [9]. Moreover, eosinophilic AECOPD events demonstrated a more stable pattern with less seasonal fluctuation compared to other triggers, validating the published data for preserved exacerbation frequency of AECOPD phenotypes associated with blood eosinophilia during the pandemic [10].

The COVID-19 pandemic could have altered AECOPD admission seasonality for various reasons. First, the seasonality of COPD exacerbation is influenced by several factors: temperature and humidity changes, cardiovascular risk, environmental factors (*e.g.* air pollution), time spent indoors (vitamin D and immune activity depending on ultraviolet exposure) and specific exacerbation triggers [11]. Overall, the mechanism behind seasonality of COPD exacerbation remains complex and multifaceted. Secondly, the implementation of social distancing measures effectively mitigated droplet transmission amongst COPD patients, who are vulnerable to viral reservoirs [12]. When international travel restrictions and mandatory mask-wearing were lifted in July 2021 [13], it is possible that a subsequent delayed surge in transmission occurred in spring of the year 2022, as observed in this cohort. Lastly, findings from the prospective pre-pandemic TORCH study indicated that seasonality primarily affected the frequency of AECOPD events rather than their severity [14], with a similar proportion of exacerbation admissions (18%) during summer and winter. Furthermore, the TIOSPIR study [15] and So *et al.* [16] also highlighted a peak in severe AECOPD cases in warmer months. Therefore, despite the pandemic precautionary measures, increased telemedicine usage and health-conscious behaviour [17], patients may still seek hospital treatment during warmer months. Further observational studies investigating the seasonal dynamics of AECOPD admissions in the upcoming years are in demand.

Our study has several limitations. Participants were recruited during their first presentation admission due to AECOPD and their subsequent readmissions were not analysed. This may introduce recruitment bias as severe cases may decline participation and repeat admissions may influence the consistency of triggers and potentially LOHS. However, it is less likely to reverse the whole seasonal pattern. Besides this, identifying triggers can be challenging in cases with viral exacerbations with superimposed bacterial infection, and some cases had overlapping characteristics. For instance, 15 patients showed viral PCR positivity and also eosinophilia on admission. As viral infection was the driver of hospitalisation and eosinophilic inflammation, those cases were categorised under viral triggers. Overall, we employed pragmatic criteria to identify triggers, mirroring those used in clinical practice, and the majority had extensive viral panel testing.

In summary, the seasonal pattern of AECOPD admissions had not returned to its usual trend [1] following 2 years of the COVID-19 pandemic. Factors such as evolving viral variants with an increasing prevalence of viral PCR testing, changes in patients' healthcare-seeking behaviours and lingering effects of pandemic-related interventions may continue to affect seasonal AECOPD patterns. Thus, it is crucial to adjust our approach to evaluating exacerbations and foster preventative measures accordingly, to alleviate the COPD-related healthcare utilisation burden. This might include enhanced surveillance during traditionally low-risk seasons for AECOPD and targeted measures to mitigate infection triggers. This finding calls for further examination of how future seasonal exacerbation patterns hold.
